# Effect of Application Deviations on Dentin Sealing of a Universal Adhesive: Permeability and Nanoleakage

**DOI:** 10.1055/s-0042-1745767

**Published:** 2022-07-11

**Authors:** Alexandre Cavalheiro, Joana Cruz, Bernardo Sousa, Ana Silva, Raquel Eira, Catarina Coito, Manuela Lopes

**Affiliations:** 1 Department of Operative Dentistry, Faculty of Dental Medicine, Universidade de Lisboa, Portugal; 2 Faculty of Dental Medicine, Universidade de Lisboa, Portugal

**Keywords:** dentin tubule sealing, etch-and-rinse, nanoleakage, self-etch, TEM, universal adhesives

## Abstract

**Objective** The objective of this study was to evaluate the effect that deviations from the recommended protocol of a universal adhesive system, applied to dentin according to the self-etch (SE) and the etch-and-rinse (ER) techniques, has on permeability and nanoleakage.

**Materials and Methods** *Permeability* : 60 extracted non-carious human third molars ( *N* = 60) were sectioned to obtain 0.7-mm-thick dentin disks. The specimens were randomly assigned to three subgroups and treated with a universal adhesive system (Prime&Bond Active Universal) using the SE and ER techniques: (1) following the manufacturer's instructions with 5 seconds drying (MFR DRY 5S), (2) following the MFR DRY 10S, and (3) reduced application time of the adhesive to 5 seconds (APPL 5S). *Nanoleakage* : 12 additional 0.7-mm-thick dentin disks were prepared, treated and divided into six groups. They were immersed in 50 wt% ammoniacal silver nitrate and processed according to conventional methods for the analysis of nanoleakage under transmission electron microscopy.

**Statistical Analysis** The results were statistically analyzed by two-way analysis of variance and *post-hoc* Bonferroni's test.

**Results** Significant differences in permeability reduction were observed among the treatment groups (0.001). The results obtained for APPL 5S were significantly lower than the results obtained for both the MFR DRY 5S ( *p* = 0.003) and MFR DRY 10S ( *p* = 0.001).

**Conclusions** The reduced application time to 5 seconds creates imperfect dentin tubule sealing, which may explain clinical reports of postoperative sensitivity and early degradation of the resin–dentin interface.

## Introduction

Over the years, adhesive systems have been studied with the purpose of improving adhesion and making clinical practice more simplified. Considered to be the latest generation of adhesive systems, multi-mode or universal adhesives can be used with a self-etch (SE), an etch-and-rinse (ER), or a selective-etch technique. 1

The ER technique not only is a multi-step protocol but also requires an initial etching step with phosphoric acid (35–37%) to remove the smear layer and to open the dentin tubules which increase dentin permeability that may not be fully reduced after the adhesive application. 2 3 On the contrary, the SE adhesives require only two steps or a single step depending on whether the adhesive system components (etchant, primer, and bonding resin) are combined in two bottles or a single bottle. 4 Consequently, with regard to the mode of application, SE adhesive systems seem to be less technique sensitive, meaning that there is a lower possibility of iatrogenically introducing clinical application mistakes. 5

Most published studies on universal adhesive systems apply them according to the manufacturer's instructions. However, clinicians may introduce deviations and mistakes while applying the adhesives in daily practice with the intention to save time. Some deviations can result in insufficient hybridization and sealing of the dentin tubules 6 7 and, consequently, hydrolyses of the collagen and degradation of the resins, 8 9 leaching of resin components, 10 increased risk of dentin permeability, 11 dentin fluid movement and nanoleakage, 12 13 and postoperative sensitivity. 14

Prati and Pashley 15 found a significant correlation between dentin permeability and bond strength of the restoration for some adhesives systems. It seems that the dentin adhesive systems with low capacity to seal dentin also have low bonding capacity. For these reasons, the studies of permeability characteristics of dentin with their interactions with the adhesive systems are of considerable physiopathological and clinical interest to explain some of the reasons for restorative failure or postoperative dentin sensitivity. 16

Considering that universal adhesive systems have different application modes, i.e., SE and ER, it is necessary to clarify the effect of simulated application deviations on dentin permeability and nanoleakage, regardless of the application modes, and, thus, on the quality of dentin sealing and potential postoperative sensitivity.

Therefore, the purpose of this study was to evaluate the reduction in dentin permeability and nanoleakage of a resin–dentin interface made with a universal adhesive, applied to dentin according to the SE technique and the ER technique, introducing deviations in the application protocol defined by the manufacturer. The null hypotheses tested were that there are no differences in adhesive dentin sealing capacity between (1) two different drying times of the adhesive in the SE and ER techniques and (2) two different application times of the adhesive in the SE and ER techniques.

## Materials and Methods

### Specimen Preparation for Permeability Study

Prior to preparation, 60 ( *N* = 60) recently extracted human third molars, intact and without evidence of caries or restorations, were randomly selected from a group of teeth, stored in 0.5% chloramine-T (Sigma-Aldrich Chemical Co., St. Louis, MO, United States) at 4°C for 1 week, and then left in distilled water at 4°C, according to the ISO TR 11405 standard. The teeth were gathered after obtaining informed consent under a protocol reviewed and approved by the Ethics Committee (Ethic No: 202001), College of Dentistry, Universidade de Lisboa.

The teeth were sectioned 2 mm below the cementoenamel junction and then in the middle third of the crown to obtain a dentin disk containing at least 0.7-mm deep dentin (IsoMet; Buehler, Lake Bluff, IL, United States). The specimen thickness was measured with a digital micrometer (Micro 2000, Moore & Wright; 0–25 mm, Sheffield, UK).

The pulpal surfaces were prepared with a diamond bur to gently remove the pulp tissue, which created a smear layer. Then, the pulpal surfaces of the specimens were conditioned with 37% phosphoric acid gel (Total Etch, Ivoclar Vivadent, Schaan, Liechtenstein) for 1 minute to completely remove the smear layer and smear plugs, opening all the tubules and, thus, allowing the fluid to freely flow within the dentin tubules during permeability measurements. 17 18

The dentin disks were then fixed on standard acrylic pieces (1 cm × 0.5 cm × 1 cm) with an impression compound. These acrylic pieces have a central channel that allows the passage of an 18G needle connected to a hydraulic system. To produce a uniform smear layer in accordance with the ISO TR 11405 standard, the dentin surface was ground with a 600 grit SiC paper (Carbimet Grit 600/P1200; Buehler, Lake Bluff, IL, United States) for 60 seconds under water irrigation.

Each specimen was connected to a hydraulic pressure system, with 37 cmH _2_ O, which is close to the normal pulpal pressure. 19 20 The fluid flow was measured by following the movement of an air bubble trapped within a glass capillary tube (0.7-mm inside diameter) (Microcaps, Fisher Scientific, Atlanta, GA, United States) that was positioned between the pressure reservoir and the dentin disk. 21

The absence of fluid conductance before the exposure of the occlusal dentin was confirmed by separately attaching five intact dentin disks to the hydraulic pressure system (as described above) and observing the (absence of) fluid movement for 2 hours. 19 20

### Dentin Permeability Measurements

The dentin permeability (P) was measured for each specimen at three different points of time: (1) before etching the occlusal side; (2) after etching, which served as the baseline (Pb), and (3) at the end of adhesive polymerization (Pa).

After the baseline measurement (Pb), which assessed the maximum permeability, the adhesives were applied. In the ER technique, the adhesive was immediately applied and light-cured thereafter. In the SE technique, the smear layer had to be recreated first, and only then, the adhesive was applied and light-cured. The output of the curing light was periodically verified at >600 mW/cm ^2^ with a radiometer (Curing Radiometer P/N 10503, Kerr, Orange, CA, United States) throughout the study.

After applying the adhesive system, the progression of the air bubble was measured.

During the application of the adhesive system, the pressure was interrupted to avoid any interference with the effectiveness of the adhesive system 22 23 and the adhesive system was applied without taking the specimen out of the system. The progression of the air bubble was measured every 2 minutes over a 6-minute interval to determine the rate of saline solution flow in millimeters per minute.

### Specimen Distribution and Treatment

The specimens were treated in a random order to avoid any bias due to a particular sequencing of treatments. Thus, the 60 specimens were randomly assigned to six groups, with 10 specimens per group. Prime&Bond Active Universal (Dentsply Sirona; Konstanz, Germany) was used for all groups; the composition of the adhesive is described in Table 1 .

**Table 1:** Materials, manufacturer, and components

**Material**	**Manufacturer**	**Components**
Prime and Bond Active Universal	Dentsply Sirona; Konstanz, Germany	Bi- and multifunctional acrylate; PENTA (dipentaerythritol pentacrylate phosphate); MDP (10-methacryloyloxydecyl dihydrogen phosphate); Initiator; stabilizer; isopropanol; water.

Thus, the experimental groups are as follows.

*Group 1: Application according to manufacturer's instruction* s, *5 seconds drying time, ER technique—(MFR DRY 5S ER)*
  (1) Phosphoric acid was applied to the dentin surface rigorously for 15 seconds. (2) The occlusal surface was rinsed with water for 15 seconds. (3) The excess water was removed using a moist cotton pellet so that the surface remained shiny and visibly moist. (4) The adhesive bottle was shaken slightly. (5) Prime&Bond Active Universal adhesive was applied with a disposable applicator brush on the dentin surface. The surface was actively rubbed for 20 seconds. (6) The surface was dried for 5 seconds, beginning with a soft blow of air from a distance of approximately 10 cm (the air pressure was increased while decreasing distance, finishing at a distance of approximately 1 to 2 mm from the surface at maximum air pressure). (7) The adhesive was light-cured for 10 seconds (Elipar S10 LED Curing Light, 3M ESPE, MN, United States).
*Group 2: Reduced application time of the adhesive to 5 seconds, ER technique—(APPL 5S ER)*
  (1) Phosphoric acid was applied to the dentin surface for rigorously 15 seconds. (2) The occlusal surface was rinsed with water for 15 seconds. (3) The excess water was removed using a moist cotton pellet so that the surface remained shiny and visibly moist. (4) The adhesive bottle was shaken slightly. (5) Prime&Bond Active Universal adhesive was applied with a disposable applicator brush on the dentin surface. The surface was actively rubbed for 5 seconds. (6) The surface was dried for 5 seconds, beginning with a soft blow of air from a distance of approximately 10 cm (the air pressure was increased while decreasing distance, finishing at a distance of approximately 1 to 2 mm from the surface at maximum air pressure). (7) The adhesive was light-cured for 10 seconds (Elipar S10 LED Curing Light, 3M ESPE, MN, United States).
*Group 3: Application according to manufacturer's instruction* s, *10 seconds drying time, ER technique—(MFR DRY 10S ER)*
  (1) Phosphoric acid was applied to the dentin surface for rigorously 15 seconds. (2)The occlusal surface was rinsed with water for 15 seconds. (3)The excess water was removed using a moist cotton pellet so that the surface remained shiny and visibly moist. (4)The adhesive bottle was shaken slightly. (5) Prime&Bond Active Universal adhesive was applied with a disposable applicator brush on the dentin surface. The surface was actively rubbed for 20 seconds. (6) The surface was dried for 10 seconds, beginning with a soft blow of air from a distance of approximately 10 cm (the air pressure was increased while decreasing distance, finishing at a distance of approximately 1 to 2 mm from the surface at maximum air pressure). (7) The adhesive was light-cured for 10 seconds (Elipar S10 LED Curing Light, 3M ESPE, MN, United States).
*Group 4: Application according to manufacturer's instruction* s, *5 seconds drying time, SE technique—(MFR DRY 5S SE)*
  (1) The adhesive bottle was shaken slightly. (2) Prime&Bond Active Universal adhesive was applied with a disposable applicator brush on the dentin surface. The surface was actively rubbed for 20 seconds. (3) The surface was dried for 5 seconds, beginning with a soft blow of air from a distance of approximately 10 cm (the air pressure was increased while decreasing distance, finishing at a distance of approximately 1 to 2 mm from the surface at maximum air pressure). (4) The adhesive was light-cured for 10 seconds (Elipar S10 LED Curing Light, 3M ESPE, MN, United States).
*Group 5: Reduced application time of the adhesive to 5 seconds, SE technique—(APPL 5S SE)*
  (1) The adhesive bottle was shaken slightly. (2) Prime&Bond Active Universal adhesive was applied with a disposable applicator brush on the dentin surface. The surface was actively rubbed for 5 seconds. (3) The surface was dried for 5 seconds, beginning with a soft blow of air from a distance of approximately 10 cm (the air pressure was increased while decreasing distance, finishing at a distance of approximately 1 to 2 mm from the surface at maximum air pressure). (4) The adhesive was light-cured for 10 seconds (Elipar S10 LED Curing Light, 3M ESPE, MN, United States).
*Group 6: Application according to manufacturer's instruction* s, *10 seconds drying time, SE technique – (MFR DRY 10S SE)*
  (1) The adhesive bottle was shaken slightly. (2) Prime&Bond Active Universal adhesive was applied with a disposable applicator brush on the dentin surface. The surface was actively rubbed for 20 seconds. (3) The surface was dried for 10 seconds, beginning with a soft blow of air from a distance of approximately 10 cm (the air pressure was increased while decreasing distance, finishing at a distance of approximately 1 to 2 mm from the surface at maximum air pressure). (4) The adhesive was light-cured for 10 seconds (Elipar S10 LED Curing Light, 3M ESPE, MN, United States).

#### Calculations to Determine Dentin Permeability

Two measurements were used to calculate, as a ratio, the dentin permeability reduction: (1) after etching (P _B_ ) and (2) after the polymerization of the adhesive (P _A_ ). The (P _B_ ) (baseline) value of dentin permeability was initially assigned as 100%. The (P _A_ ) value of dentin permeability was expressed as a percentage of this maximum value [100 − (P _A_ /P _B_ × 100)]. Thus, each specimen served as its own control.

### Specimen Preparation for Nanoleakage

Twelve additional 0.7-mm-thick dentin disks (two for each of the six adhesive groups) were prepared, and adhesives were applied in a manner similar to the one used for the permeability measurements. After storing them in distilled water at 37°C for 24 hours, a 1-mm-wide slab containing the resin–dentin interface was prepared from the widest portion of each bonded disk. The slabs were immersed in 50 wt% ammoniacal silver nitrate solution in the dark for 24 hours, 24 without allowing them to dehydrate, and prepared for nanoleakage. After the reduction of the diamine silver ions, the silver-impregnated slabs were processed for transmission electron microscope (TEM) examination without demineralization. Ninety nanometer-thick epoxy resin-embedded sections were prepared and examined unstained, 24 using a TEM (FEI Tecnai G2 Spirit BioTWIN, operating at 120 keV, equipped with an Olympus-SIS Veleta CCD camera). The images were compared and descriptively analyzed.

## Statistical Analysis

Sample size calculations were performed using the G*Power Program Statistical Analysis, 25 26 with *α* = 0.05, the desired power of 80%, and data from the pilot study.

For the dentin permeability study, the results were statistically analyzed by two-way analysis of variance and *post-hoc* Bonferroni's test (IBM SPSS Statistics for Windows, Version 23.0; IBM Corp., Armonk, NY, United States) to evaluate the effects of the mode of application and the deviations from the recommended protocol for clinical use on the reduction in dentin permeability.

## Results

### Dentin Permeability Study

The Kolmogorov–Smirnov test of normality at α = 0.05 revealed that the data presented a normal distribution, and Levene's test was used to verify the homogeneity of the variances ( *p* = 0.357), enabling a parametric analysis.

**Fig. 1:**
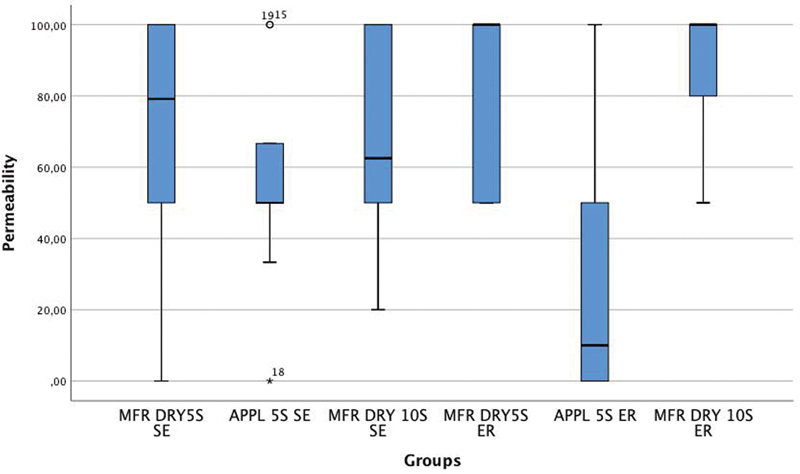
Box-and-whisker plots of percentage permeability reduction.

The data on percent permeability reduction are summarized in Table 2 and Fig. 1 , by deviations from the recommended protocol for clinical use and application modes as mean and standard deviation (SD) are also presented.

**Table 2:** Data description of percentage permeability reduction (%)

**Deviations from the recommended protocol for clinical application**	**Application mode**	**Mean ± SD (%)**
MFR	ER	80 (25.8)
	SE	70.8 (33.2)
APPL 5S	ER	28.7 (35.9)
	SE	56.7 (29.6)
DRY 10S	ER	88.0 (20.9)
	SE	67.8 (30.9)

Abbreviations: APPL 5S, application time of the adhesive to 5 seconds; DRY 10S, 10 seconds drying; ER, etch-and-rinse; MFR, manufacturer's instructions; SD, standard deviation; SE, self-etch.

MFR DRY 10S ER had the largest mean (88.0%) and APPL 5S ER had the lowest mean (28.7%).

Table 3 lists the *p* -values. No differences in permeability reduction were observed between the two application modes (SE*ER) ( *p* = 0.954). However, significant differences in permeability reduction were observed among the study groups (0.001). The results obtained for MFR DRY 5S were significantly higher than those obtained for APPL 5S ( *p* = 0.003), and the results obtained for MFR DRY 10S were significantly higher than those obtained for APPL 5S ( *p* = 0.001).

**Table 3:** Results from two-way analysis of variance (ANOVA) and *post-hoc* Bonferroni's test

**Deviations from the recommended protocol for clinical application**	**Deviations from the recommended protocol for clinical**	**Sig**
MFR (SE + ER)	APPL 5S (SE + ER)	0.003
	DRY 10S (SE + ER)	1.000
APPL 5S (SE + ER)	MFR (SE + ER)	0.003
	DRY 10S (SE + ER)	0.001
DRY 10S (SE + ER)	MFR (SE + ER)	1.000
	APPL 5S (SE + ER)	0.001
SE*ER		0.954

Abbreviations: APPL 5S, application time of the adhesive to 5 seconds; DRY 10S, 10 seconds drying; ER, etch-and-rinse; MFR, manufacturer's instructions; SD, standard deviation; SE, self-etch.

### Nanoleakage Study

**Fig. 2:**
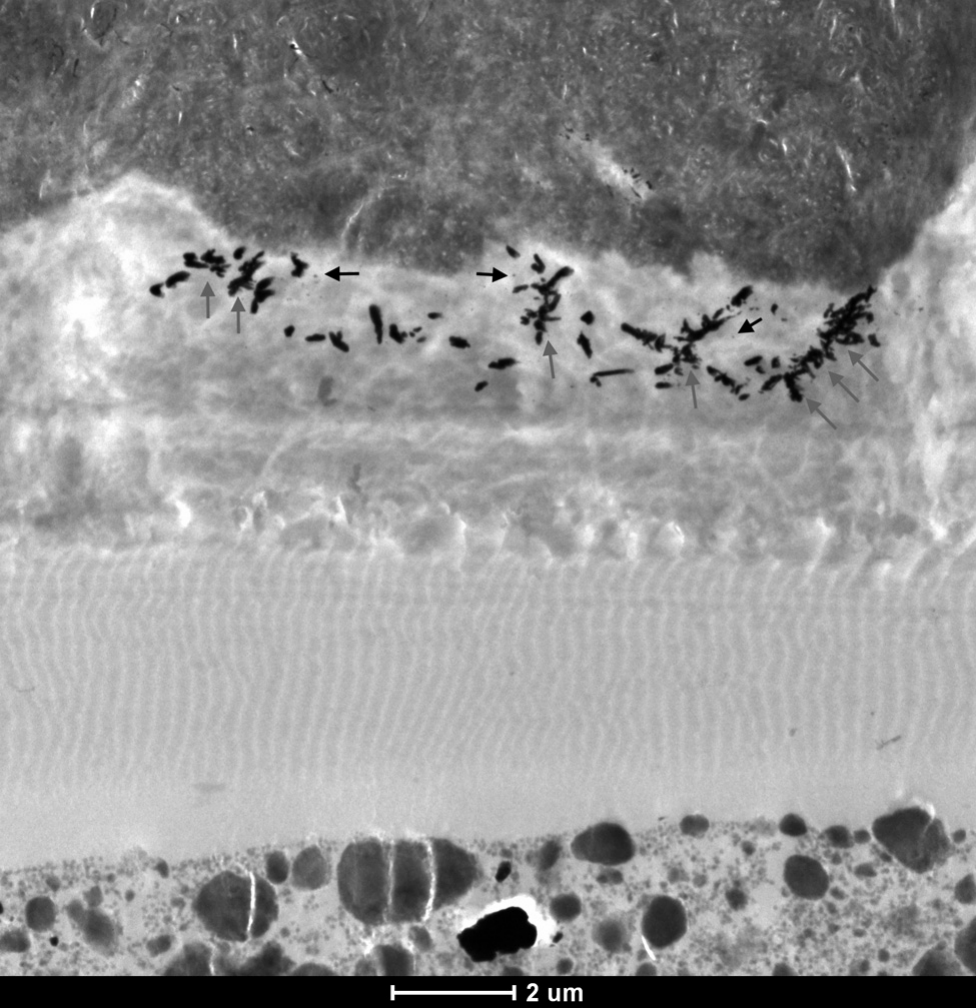
TEM micrograph of representative area of unstained, undemineralized, and silver-impregnated sections of the MFR DRY 5S ER group. 6,000x.

In the MFR DRY 5S ER group, two types of nanoleakage patterns could be observed within the resin–dentin interfaces: spotted ( *black arrows* ) and reticular ( *gray arrows* ) patterns as shown in Fig. 2 . The spotted pattern consisted of isolated spots of silver grains that were observed in the hybrid layer in various amounts. The reticular pattern consisted of discontinuous islands of silver deposits exclusively observed in the hybrid layers or in the hybridized areas of resin tags.

**Fig. 3:**
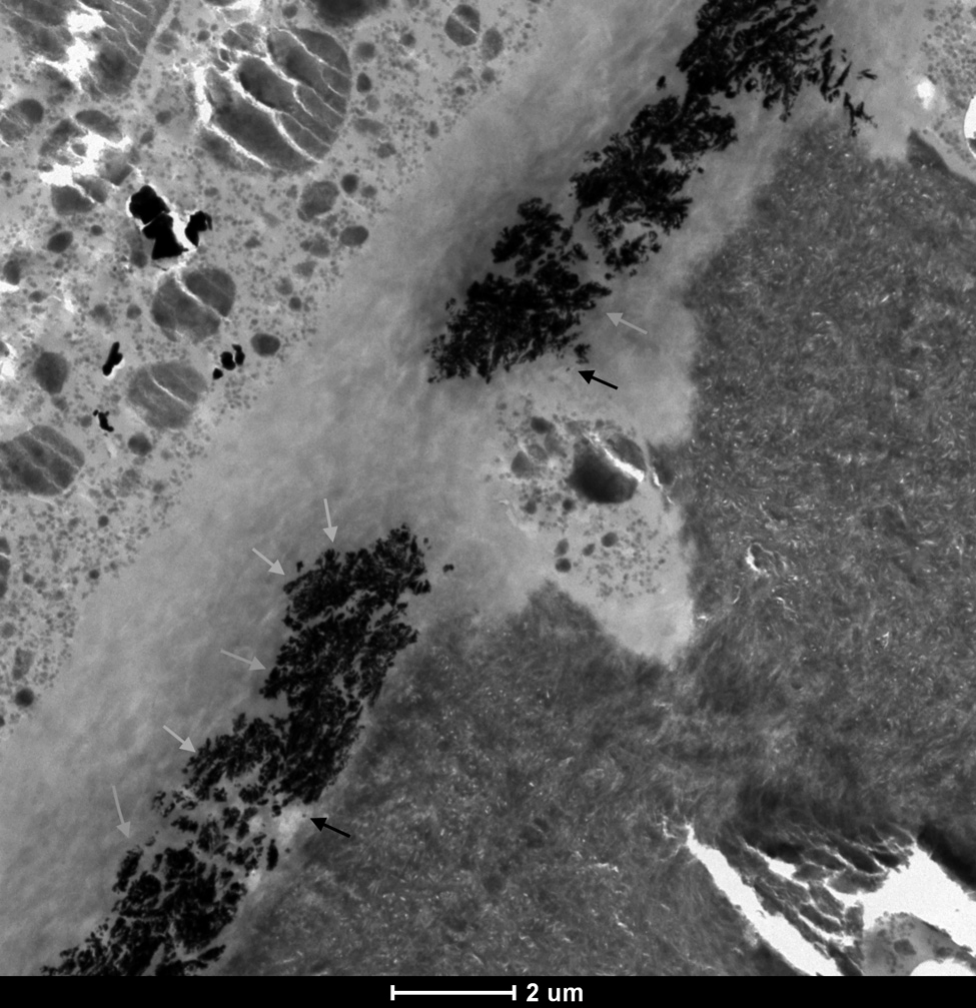
TEM micrograph of representative area of unstained, undemineralized, and silver-impregnated sections of the APPL 5S ER group. 6,000x.

In the APPL 5S ER group, the hybrid layer was impregnated with an extensive reticular type ( *gray arrows* ) and spotted type ( *black arrows* ) of nanoleakage, as illustrated in Fig. 3 . The severity of nanoleakage in this group was so high that the reticular silver deposits not only occurred continuously along the entire length of the hybrid layer but also extended to its entire thickness.

**Fig. 4:**
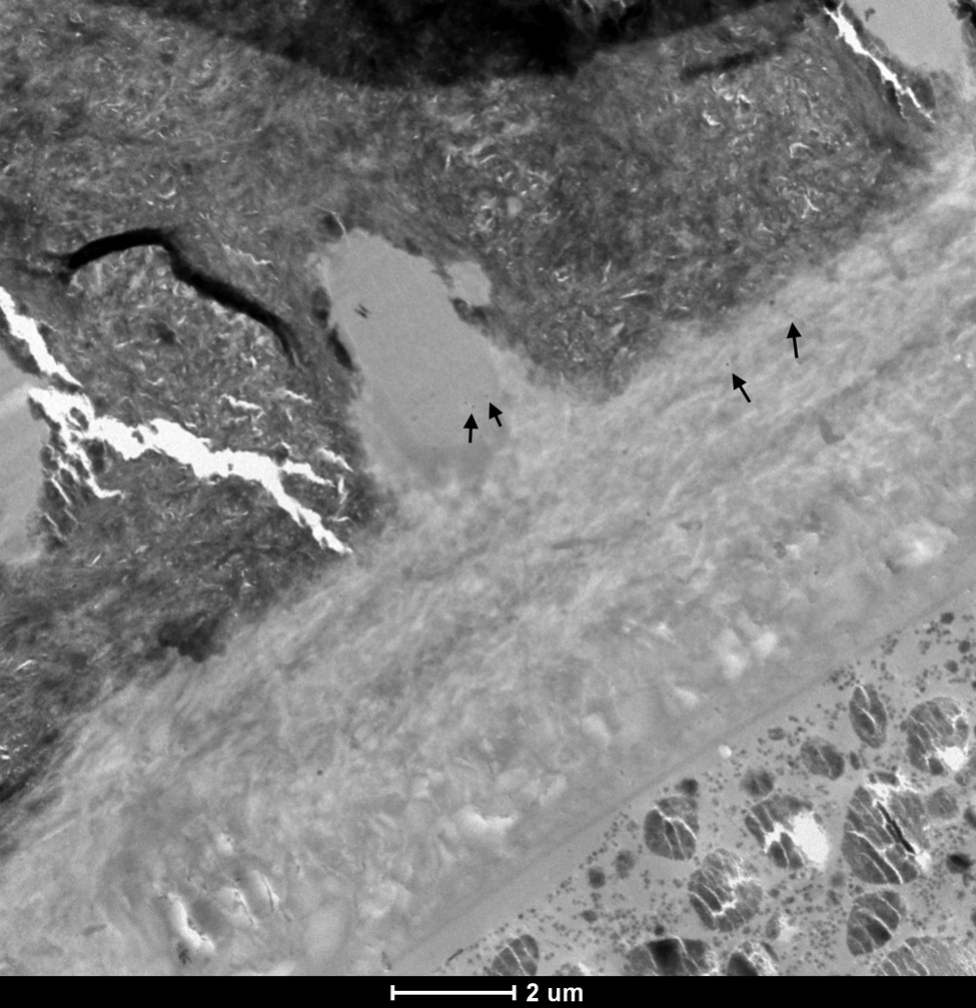
TEM micrograph of representative area of unstained, undemineralized, and silver-impregnated sections of the MFR DRY 10S ER group. 6,000x.

The MFR DRY 10S ER group did not show significant nanoleakage, and isolated spots of silver grains ( *black arrows* ) could only be discerned at high magnification ( Fig. 4 ).

**Fig. 5:**
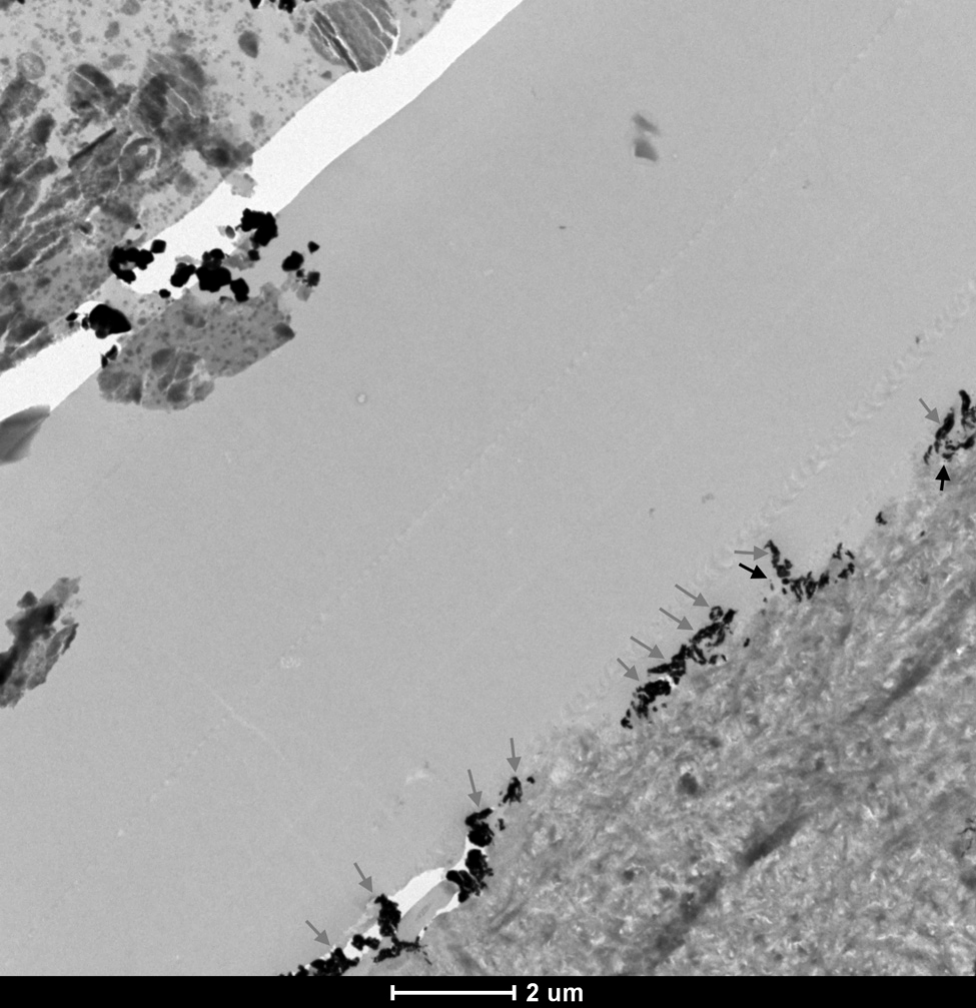
TEM micrograph of representative area of unstained, undemineralized, and silver-impregnated sections of the MFR DRY 5S SE group. 6,000x.

The presence of a linearly distributed reticular nanoleakage pattern along the base was observed in the MFR DRY 5S SE group. A spotted type of nanoleakage ( *black arrows* ) was also noted, as illustrated in Fig. 5 .

**Fig. 6:**
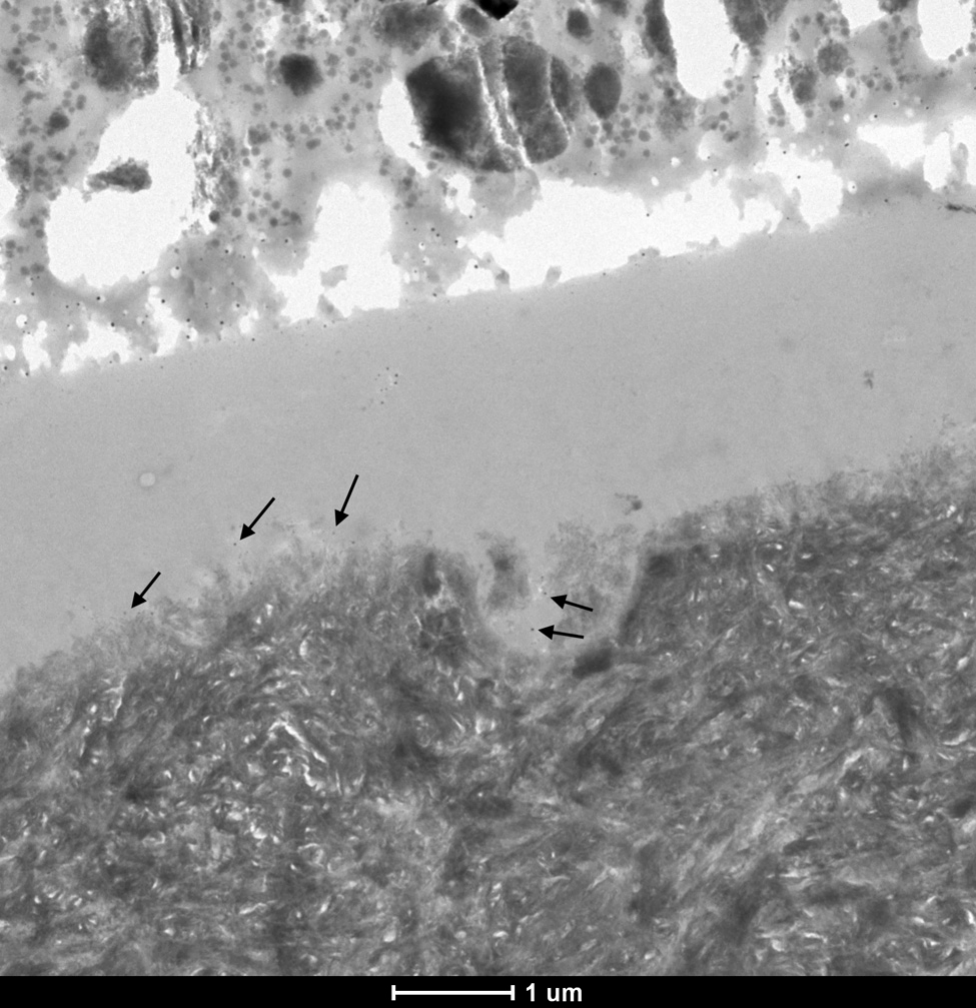
TEM micrograph of representative area of unstained, undemineralized, and silver-impregnated sections of the MFR DRY 10S SE group. 11,500x.

The MFR DRY 10S SE group did not show any significant nanoleakage. Isolated spots of silver grains ( *black arrows* ) that were distributed in the hybrid layer were noted, but they could only be discerned at high magnification ( Fig. 6 ).

**Fig. 7:**
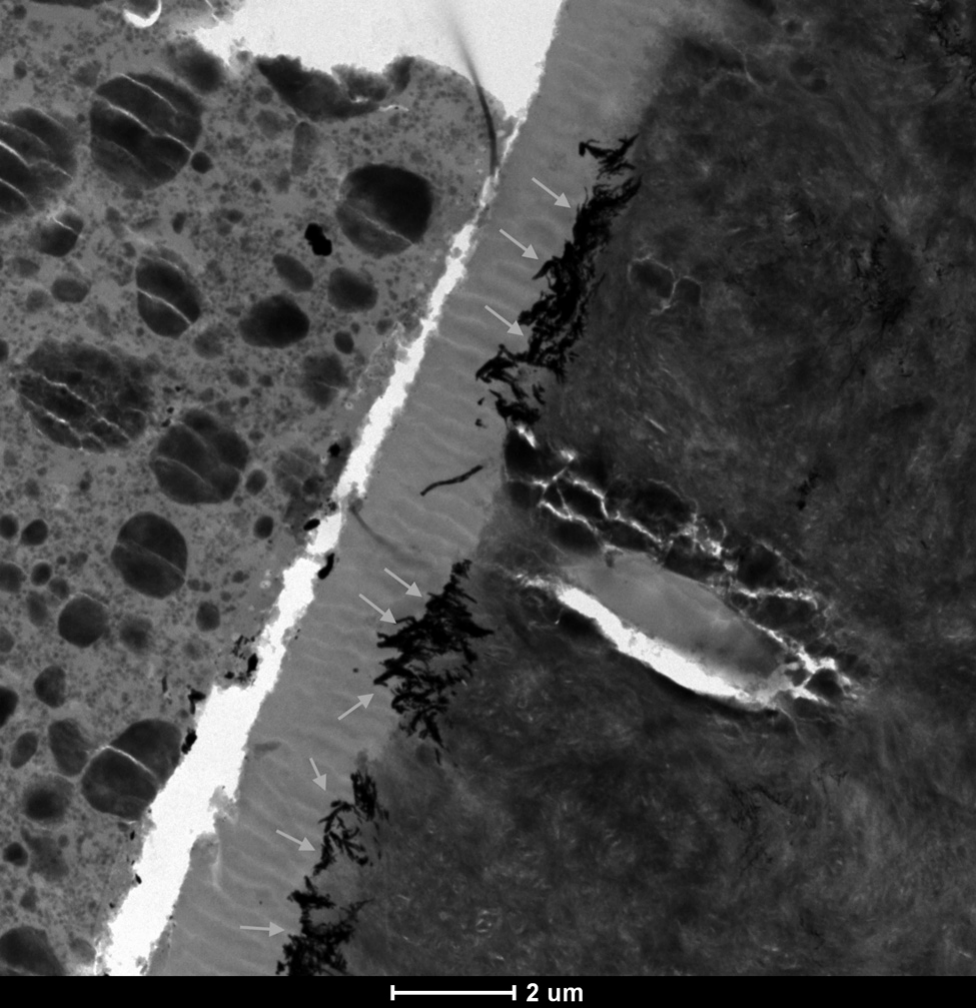
TEM micrograph of representative area of unstained, undemineralized, and silver-impregnated sections of the APPL 5S SE group. 6,000x.

In the APPL 5S SE group, silver deposits were also agglomerated in a reticular pattern ( *gray arrows* ) at the base of the hybrid layer, as illustrated in Fig. 7 .

## Discussion

Universal adhesive systems emerged in an attempt to facilitate the clinical procedure, allowing the reduction of the application time but, most importantly, allowing a greater versatility on the mode of application and adhesive substrates, leading to them being increasingly used by dentists. 1 For this reason, it is important to understand how best to apply them and the mistakes that should be avoided during their application so that adhesion is not compromised.

Considering that dentin sealing is one of the main objectives of adhesive systems, the study of dentin permeability allows us to understand the effectiveness of adhesion because a significant correlation between bond strength and dentin permeability of the restoration for some adhesive systems has been demonstrated, that is, it is now known that adhesives with low sealing capacity have low adhesive capacity. 15

A hydraulic conductance study has advantages over other types of leakage studies 27 because it allows repeated measurements on the same specimen longitudinally and non-destructively, gives a quantitative measurement of the interfacial leakage, assesses whether the dentin tubules are effectively sealed, and finally, measures dentin permeability at the baseline (after etching) and after the adhesive polymerization, allowing each specimen to serve as its own control.

Ideally, adhesive systems are expected to reduce the dentin permeability completely. In this study, the adhesive system for any of the groups was not capable of completely reducing the dentin permeability produced after etching, and all groups presented at least one nanoleakage pattern, irrespective of the application technique used. These nanoleakage results seem to indicate that this universal adhesive behaves similarly to conventional two-step ER or single-step SE adhesives that are always permeable to water at different levels. 28 29 In fact, hydrophilic polymers, 1 30 like the ones used in the adhesive of this study, function as permeable membranes that allow the movement of water through the dentin even after polymerization. This seems to indicate that the use of this universal adhesive may present clinically postoperative sensitivity, regardless of the mode of application—SE or ER. 31

The universal adhesive system Prime&Bond Active Universal has a pH >2.5 and is, therefore, considered a high-pH adhesive. Studies show that, in adhesive systems with a higher pH, 32 the previous application of phosphoric acid has additional beneficial effects. 32 33 In this study, although there are no differences between the application modes, the permeability reduction in the MFR and DRY 10S groups was slightly higher in the ER application mode than in the SE application mode, which is in line with the results obtained in other studies. 32 33

Some studies 6 22 show that the occurrence of errors or deviations from the application protocol defined by the manufacturer results in inconsistent adhesion forces, compromising the performance of the adhesive system.

In the APPL 5S ER group, the permeability after applying the adhesive is similar to the maximum permeability, with a permeability reduction of only 28.7%. The APPL 5S SE group also achieved only a 56.7% permeability reduction. The reduction in the application time of the adhesive, although very appealing to clinicians, may imply a compromise in infiltration and evaporation of the solvent, leading to lower adhesion forces and an acceleration in the degradation process of the adhesive interface. 34 35 Therefore, according to the results of this study, it is recommended to carefully follow the manufacturer's instructions regarding the application time of the adhesive system.

The drying of the adhesive is a crucial step to guarantee good adhesion results, as it allows the evaporation of solvents, preventing weak polymerization, dilution, and phase separation of the different constituents. 22 The best result was obtained by the DRY 10S ER group, which may mean that in the ER technique, an increase in drying time from 5 seconds to 10 seconds improves adhesion.

Similarly to older generations of adhesives, 36 the results of this study seem to indicate the importance of sufficient application time and careful drying of universal adhesives, regardless of the application technique (ER or SE). 1 22

This study has some limitations. It was not possible to control some variables related to the tooth itself, such as regional differences in dentin permeability, 37 aging, 38 or dentin sclerosis. 39 To standardize the sample, the specimens used consisted of caries-free teeth; however, in clinical practice, adhesive procedures are often performed on teeth affected by caries lesions. 40

The evaluation of the clinical performance of adhesive systems should include conducting clinical trials, as *in vitro* studies do not allow for the correct assessment of all variables associated with clinical practice. Thus, it is not possible to make a direct extrapolation of the results obtained in this laboratory study to clinical situations. Yet, it can be expected that the existence of statistically significant differences has a higher probability of corresponding to clinically significant differences than their absence. Additional clinical studies are essential to further evaluate the performance of universal adhesives and the impact of the adhesive application deviations on the hydrolytic degradation rates of the resin–dentin bond over time.

## Conclusion

The results of this study require the rejection of the null hypotheses. The manufacturer's protocol must comply with regard to the application time of the adhesive. The reduction of the application time to 5 seconds results in a lower reduction of permeability values.
